# Editorial for the Special Issue “Recent Advances in Nanomaterials Science”

**DOI:** 10.3390/ijms25105541

**Published:** 2024-05-19

**Authors:** Gang Ho Lee

**Affiliations:** Department of Chemistry, College of Natural Sciences, Kyungpook National University, Daegu 41566, Republic of Korea; ghlee@mail.knu.ac.kr

Nanoparticles and nanomaterials are important, because they are potentially applicable to energy, storage, bioimaging, biosensors, catalysts, nanomedicine, batteries, solar energy, bioenergy, and so on (Figure 1) [1,2,3,4]. This Special Issue presents research on a variety of nanoparticles and nanomaterials, including organic, inorganic, polymeric nanoparticles and nanomaterials, biomolecules, and bio-nanomaterials.

The synthesis and characterization of nanoparticles and nanomaterials are also very important. The physicochemical properties of nanoparticles and nanomaterials can be engineered through the control of the size, shape, composition, and so on. Therefore, the development of new synthesis and characterization methodologies is important to invent new nanoparticles and nanomaterials with desired properties. In addition, for the wide application of nanoparticles, their toxicity and environmental effects should be carefully considered and minimized [5].

Twenty-three state-of the art articles (20 original papers and 3 reviews) on nanoparticles and nanomaterials from experts across the globe were accepted for this Special Issue. Nanoparticles can be used for the removal/recycling of heavy metal ions in aqueous media, as their adsorption efficiency is very high (>80%). The nanoparticles can be reused through desorption of the metal ions in an acidic medium. Photoluminescent (PL) nanoparticles are important, as they can be applied to electronic displays, light sources, and bioimaging. Metal nanoparticles can be used as catalysts. Their catalytic activity depends on the surface conditions of the nanoparticles. Metal nanoparticles can be reused for catalytic reaction after magnetic separation. Nanoparticles can be synthesized using various methods. They can be prepared in a gas phase (i.e., in a vacuum), under atmospheric conditions, and in solution. For commercial applications, large-scale synthesis is needed. For example, mechanical milling is undoubtedly a promising approach to develop a scalable synthesis of Cu-based particles, with potential applications in various fields such as electronics, water remediation, sensors, pharmaceutical sciences, packaging, anti-microbial materials, and agriculture. 

There is no doubt that nanoparticles and nanomaterials are promising materials for future applications to various fields (Figure 1), due to their unique physicochemical properties, which are different to those of atoms, molecules, and bulks. To realize this, continuous research should be conducted. 

## Figures and Tables

**Figure 1:**
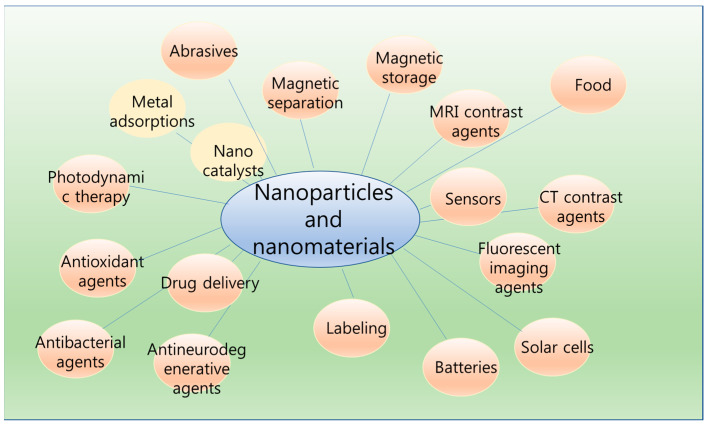
Various application fields of nanoparticles and nanomaterials.
